# Hypertension and Obesity in Dakar, Senegal

**DOI:** 10.1371/journal.pone.0161544

**Published:** 2016-09-13

**Authors:** Enguerran Macia, Lamine Gueye, Priscilla Duboz

**Affiliations:** 1 UMI 3189 Environnement, santé, sociétés (CNRS / Université Cheikh Anta Diop / Université de Bamako / CNRST Burkina-Faso), Faculté de Médecine, de Pharmacie et d’Odontostomatologie, Dakar, Senegal; 2 UMR 7268 Anthropologie, Droit, Ethique et Santé (CNRS / Aix-Marseille Université / EFS), Faculté de Médecine, Secteur Nord, CS 80011, Marseille Cedex 15, France; Institute of Infectious Diseases and Molecular Medicine, SOUTH AFRICA

## Abstract

**Background:**

Cardiovascular disease is a major public health problem in many sub-Saharan African countries, but data on the main cardiovascular risk factors–hypertension and obesity–are almost nonexistent in Senegal. The aims of this study were therefore (i) to report the prevalence, awareness, treatment and control of hypertension among adults in Dakar, (ii) to assess the prevalence of general and central obesity, and (iii) to analyze the association between hypertension and general and central obesity.

**Methods:**

A cross-sectional survey was carried out in 2015 on a representative sample of 1000 dwellers of the Senegalese capital aged 20–90.

**Results:**

The overall prevalence of hypertension was 24.7%. Among hypertensive respondents, 28.4% were aware of their condition; 16.0% were on antihypertensive medication; 4.9% had controlled blood pressure. The frequency of doctor visits was a significant predictor of awareness (OR = 2.16; p<0.05) and treatment (OR = 2.57; p<0.05) of hypertension. The prevalence of underweight, overweight and general obesity were 12.6%, 19.2% and 9.7% respectively. The prevalence of central obesity was 26% by WC and 39.8% by WHtR. General obesity and central obesity by WHtR significantly predicted HTN among men and women, but not central obesity by WC.

**Conclusions:**

This study has demonstrated a high prevalence of hypertension in Dakar and a high prevalence of obesity among women–particularly among older women. The awareness, treatment, and effective control of hypertension are unacceptably low. The blood pressure of women with general obesity, and men with central obesity, in the community should be monitored regularly to limit the burden of cardiovascular disease in Senegal.

## Introduction

Cardiovascular disease (CVD) has become the leading cause of death worldwide [1,2], and is now a major public health problem in low and middle-income countries [3]. About 80% of the global burden of CVD deaths already occurs in these countries [4]. In Africa, the age-specific mortality rates from CVD are much higher in younger age groups in both men and women than in the developed world [5]. Sub-Saharan Africa (SSA) is currently facing the epidemiologic [6,7] and nutrition [8] transitions; and Senegal is among the SSA countries at the most advanced stage within these transitions [9]. By the year 2030, the burden of cardiovascular disease in SSA is expected to nearly double [10,11], however, reliable data on the main cardiovascular risk factors–hypertension (HTN) and obesity–from Africa are scarce [12,13], particularly in francophone West Africa [14].

HTN is the major driver of the CVD epidemic in SSA, where it is an independent risk factor for heart failure and stroke [15]. As many recent reviews have demonstrated [14, 16–20], HTN is already a massive health problem in SSA [21]. The prevalence of HTN in the region varies widely from study to study, ranging from 7.5% in a population of Sudanese young adults (mean age 35 years; in 1990) [22], to over 77% among older adults in South Africa (mean age 65 years; in 2008) [23]. According to the meta-analysis made by Altalke et al. [14], the prevalence of HTN in SSA at mean participant ages of 30, 40, 50, and 60 years are 16%, 26%, 35%, and 44%, respectively, with a pooled prevalence of 30%. While this overall prevalence is similar to what can be observed in the United States for instance [24], it is above all the very low rates of awareness, treatment and control noted in SSA that are cause for concern [25]. Again according to this same meta-analysis, 27% of hypertensive people were aware of their hypertensive status, 18% were receiving treatment, and only 7% had controlled blood pressure. In Senegal, only one recent research, limited in scope, has been conducted on this issue among the adult population living in Dakar, yielding similar figures: in 2009, prevalence of HTN, awareness, treatment and control were 27.50%, 27.88%, 16.97% and 5.45%, respectively [26,27].

Overweight and obesity are also important risk factors for CVD [28,29]. In urban West Africa, the prevalence of obesity more than doubled from 7.0% in 1990–94 to 15.0% in 2000–04 [30], despite the continued burden of undernutrition [31]. The co-existence of problems of excess weight combined with those of underweight–a condition termed the ‘double burden’ [32]–well illustrates the difficulties produced by the nutrition transition in SSA. To date, a single study has been carried out in Dakar on weight problems among the general adult population, indicating that in 2009, in terms of body mass index (BMI), the prevalence of underweight, overweight and general obesity were 12.3%, 22.3% and 8.3%, respectively, whereas the prevalence of central obesity was 21.2% using waist circumference (WC) [33].

Moreover, the relationship between obesity, including both general obesity and central obesity, and HTN has been observed in many studies [34–37] and is known to vary among populations and ethnicities [38]. However, to our knowledge, this relation has never been specifically studied in Senegal. Therefore, the aims of this study were (i) to report the prevalence, awareness, treatment and control of HTN among adults in Dakar, (ii) to assess the prevalence of general and central obesity, and (iii) to analyze the association between HTN and general and central obesity.

## Materials and Methods

### Ethics statement

The study was approved by the National Ethics Committee for Health Research of Senegal (protocol SEN13/67, n°0272). This research has been conducted in accordance with the declaration of Helsinki. Written informed consent was obtained from participants. People diagnosed with HTN were referred to health units.

### Population sample

This study was conducted from February to August 2015 on a sample of 1,000 individuals aged 20 and older. The sample was constructed using the combined quota method [39] (cross-section by age, gender and town of residence) in order to strive for representativeness of the population aged 20 and older living in the department of Dakar. Data from the *Agence Nationale de la Statistique et de la Démographie* dating from the last census (2013) were used. According to the last census, the population of the department of Dakar was 1,146,053 in 2013, including 725,373 adults aged 20 and over. The quota variables used were gender (male/female), age (20–29 / 30–39 / 40–49 / 50–59 / 60 and over, with an upper age limit of 100 years, but concretely, the oldest participant was aged 90) and town of residence. The towns were grouped by the four arrondissements making up the department of Dakar: Plateau-Gorée (5 towns), Grand Dakar (6 towns), Parcelles Assainies (4 towns) and Almadies (4 towns). Practically, this method requires constructing a sample that reflects the proportions observed in the general population. For example, according to the last census, women aged 20–29 living in town of Medina (arrondissement of Plateau-Gorée) represented 1.5% of the population aged 20 and older living in the department of Dakar. The sample has been constructed so as to reflect this proportion and include 15 women aged 20–29 living in this town. In order to limit any bias associated with this sampling method, the investigators worked at different moments of the day (and sometimes on Saturday and Sunday) and, in each town of residence, began their investigation from different starting points each day. Pregnant women were excluded from the study.

Eight trained investigators (PhD students in Medicine, Pharmacy and Sociology) started out each day from different points in each town to interview individuals in Wolof, Haalpulaar or French in every third home, i.e. the dwelling behind every third front door or entrance gate. Investigators had a certain number of individuals to interview (women aged 20–29 / men aged 20–29 / women aged 30–39 / men aged 30–39 / women aged 40–49 / men aged 40–49 / women aged 50–59 / men aged 50–59 / women aged 60 and over / men aged 60 and over) to meet the quotas. Only one person was selected as a respondent in each home. Investigators went to the house, inquired about the inhabitants and then chose the first person they saw who met the characteristics needed for the quotas. In-person interviews were conducted. They ranged from 45 minutes to more than 1 hour and 30 minutes, depending on respondent availability and desire to talk.

### Study definitions and measurements

#### Blood pressure and hypertension

Blood pressure was measured twice for each participant in the course of a single visit. The first measurement was taken mid-way through the interview, just after the questions related to individual health. The second measurement was taken at the end of the questionnaire, i.e. after about 15–20 minutes’ rest. These measurements were taken by trained students, using an Omron® M3 Intellisense device validated by the International Protocol [40]. The mean of the two measurements was retained for the analyses. In accordance with the Seventh Report of the Joint National Committee of Prevention, Detection, Evaluation, and Treatment of High Blood Pressure, individuals with systolic blood pressure (SBP) ≥ 140 mmHg and/or diastolic blood pressure (DBP) ≥ 90 mm Hg and/or who reported the current use of antihypertensive medication were considered as suffering from high blood pressure [41]. Participants were classified as hypertensive aware if they reported that they had previously been informed by health professional that they had HTN. Hypertensive aware participants were classified as being on treatment if they reported current use of drugs prescribed by a health professional. Control was defined as systolic blood pressure < 140 mmHg and diastolic blood pressure < 90 mmHg among people treated for HTN.

#### Body mass index (BMI), waist circumference (WC), and waist-to-height ratio (WHtR)

Weight was measured using a digital scale (measurement accuracy of 100 g), with subjects dressed in minimal clothing and barefoot. To measure height, the subject was to stand “at attention,” arms at sides, heels joined, without shoes. Following World Health Organization recommendations, BMI was calculated by dividing weight (kg) by the square of the height (m^2^). Underweight was defined as BMI <18.5; overweight was defined as 25 ≤ BMI < 30; obesity corresponded to a BMI of ≥ 30 [38]. Waist circumference (WC) was measured at the thinnest point of the abdomen at the end of a normal expiration. WC was measured using a measuring tape with 1-mm accuracy. WC of ≥ 102 cm in men and of ≥ 88 cm in women was considered central obesity [42]. Waist-to-height ratio was used as another indicator of central obesity since it was demonstrated that this variable is a better predictor of cardiovascular risk than BMI and WC in SSA [43]. WHtR was calculated using the formulae: *WHtR = WC (cm)/height (cm)*; the threshold of > 0.5 was used, as recommended for Causasian, Asian and African populations [44–46]

#### Socio-demographic variables

Among the socio-demographic data collected during the interviews, three variables were taken into account for this study: age, gender, and educational level. Four age groups were defined: 20–29, 30–39, 40–49 and 50 and over. Gender was coded as follows: 1 for women, 0 for men. Five levels of education were defined, in accordance with to the Senegalese school system: none, primary (1 to 5 years of schooling) intermediate (6–8 years), secondary (9–12 years), university (13 years and over).

#### Frequency of doctor visits

Given the importance of frequency of doctor visits in explaining awareness, treatment and control of HTN [47], this variable was included in the analyses. Moreover, due to the large proportion of persons who had not visited a doctor in the year preceding the interview (45%), the frequency of doctor visits was dichotomized as in the study conducted by the hypertension study group in India and Bangladesh [48]. Thus, persons who had not visited a doctor in the year preceding the interview were distinguished from those who had seen a doctor at least once during the year.

### Analysis

To answer our research questions, we used Student t-tests, Chi-square tests, and logistic regressions. Results were expressed as mean ± standard deviation for continuous variables or as percentages for categorical variables. Bivariate comparisons were performed using Student t-tests for continuous variables, and Chi-square tests for categorical variables. Multivariate analyses were performed using binary logistic regression and results were expressed as odds ratios with 95% confidence intervals (CIs). In these binary logistic regression models, dichotomous outcome variables were: HTN, awareness and treatment; as well as general and central obesity. The software used for the statistical analysis was SPSS Statistics 22 for Windows.

## Results

Among the 1000 individuals included in the study, 16 women were finally excluded because they reported pregnancy. Analyses were performed on a sample of 984 Dakarites. The distributions of age, height, weight, BMI, WC, WHtR, SBP, DBP, sociodemographic variables, and frequency of doctor visits by gender and comparisons between males and females are summarized in Table 1. The results show that men and women differed for all the factors studied except for age, DBP, and HTN. In particular, general and central obesity were largely more frequent among women than among men; and women more often than men had seen a medical doctor at least once in the previous year (Table 1).

**Table 1:** Characteristics of the Dakar sample.

Characteristics	Total (N = 984)	Male (N = 494)	Female (N = 490)	P
Age (year)	35.70 ± 13.16	35.89 ± 13.27	35.51 ± 13.07	0.652
Height (cm)	172.56 ± 9.87	178.96 ± 8.07	166.11 ± 6.88	<0.001
Weight (kg)	69.28 ± 14.44	70.21 ± 16.67	68.34 ± 16.00	0.043
BMI (Kg/m²)	23.33 ± 4.89	21.91 ± 3.54	24.76 ± 5.59	<0.001
General obesity (n, %)	35 (9.7%)	14 (2.8%)	81 (16.5%)	<0.001
WC (cm)	84.31 ± 13.02	81.51 ± 10.65	87.14 ± 14.51	<0.001
Central obesity by WC (%)	256 (26%)	21 (4.3%)	235 (48%)	<0.001
WHtR	0.49 ± 0.08	0.46± 0.06	0.53± 0.09	<0.001
Central obesity by WHtR (%)	392 (39.8%)	101 (20.4%)	291 (59.4%)	<0.001
SBP (mmHg)	123.43 ± 17.92	126.51 ± 16.48	120.32 ± 18.77	<0.001
DBP (mmHg)	81.59 ± 11.85	81.33 ± 11.50	81.84 ± 12.19	0.507
Hypertension (%)	243 (24.7%)	120 (24.3%)	123 (25.1%)	0.768
Educational level				<0.001
None	208 (21.1%)	84 (27%)	124 (25.3%)	
Primary	348 (35.5)	163 (33%)	185 (37.8%)	
Intermediate	197 (20%)	109 (22.1%)	88 (18%)	
Secondary	91 (9.2%)	51 (10.3%)	40 (8.2%)	
University	140 (14.2%)	87 (17.6%)	53 (10.8%)	
Doctor visit in previous year				<0.001
0	442 (44.9%)	270 (54.7%)	172 (35.1%)	
1 or more	542 (55.1%)	224 (45.3%)	318 (64.9)	

BMI = body mass index; WC = waist circumference; WHtR = waist-to-height ratio; SBP = systemic blood pressure; DBP = diastolic blood pressure.

General obesity was defined as body mass index (BMI) ≥ 30 kg/m²; central obesity based on waist circumference (WC) was defined as WC ≥ 102 cm for men and ≥ 88 cm for women; central obesity based on WHtR was defined as WHtR > 0.5.

### Prevalence, awareness, treatment and control of hypertension

**Fig 1:**
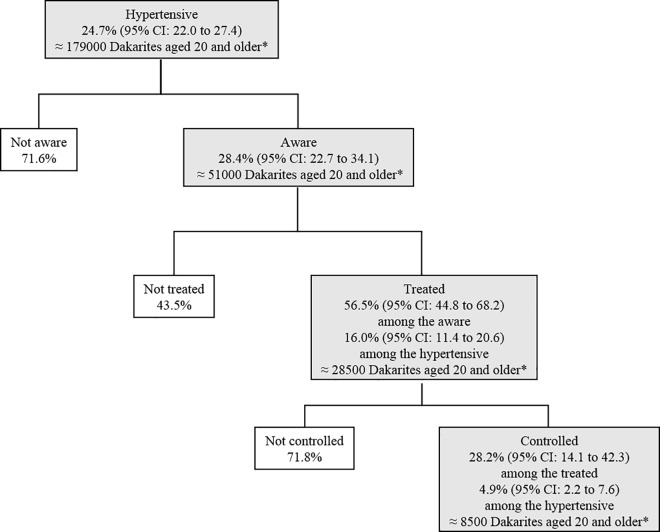
Prevalence, awareness, treatment and control of hypertension in the adult population of Dakar.

In our sample, the prevalence of HTN was 24.7% (95% CI: 22.0–27.4). Barely more than 28% of the individuals suffering from HTN were aware of their health problem, and 56.5% of the informed people reported being treated for HTN. Therefore, 16% (95% CI: 11.4–20.6) of the people suffering from HTN were treated. However, among people reporting they were treated for HTN, only 28.2% had controlled HTN; i.e. 4.9% (95% CI: 2.2–7.6) of the hypertensives (Fig 1).

**Fig 2:**
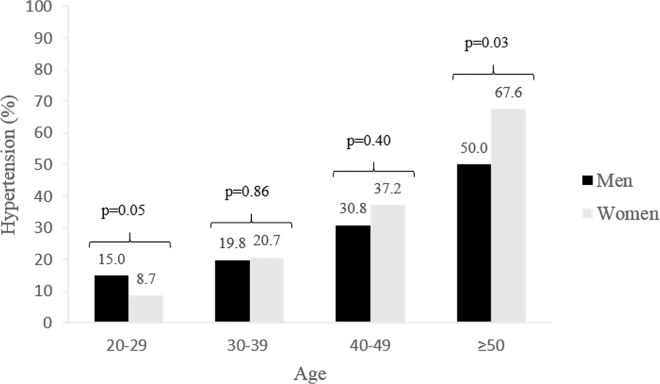
Prevalence of hypertension by age group.

Among men and women, the prevalence of HTN increases regularly with age, and except for the younger age group, women were more often hypertensive than men (Fig 2). These differences between men and women of the same age groups were significant only for younger and older adults (Fig 2). Multivariate analyses showed that age and educational level were associated with HTN (Table 2). Among hypertensives, gender, age, and the frequency of doctor visits were associated with awareness of HTN (Table 2). The small absolute number of hypertensive subjects with controlled blood pressure made it impossible to perform trend analysis on the control of HTN.

**Table 2:** Adjusted Odds Ratio for hypertension, awareness, and treatment.

Variables	Categories	Hypertension (n = 984)		Awareness (n = 243)		Treatment (n = 243)	
		OR	CI (95%)	OR	CI (95%)	OR	CI (95%)
Age (20–29)	30–39	1.70*	1.10–2.61	5.8*	1.16–28.96	6.57	0.68–63.68
	40–49	3.47***	2.20–5.46	6.98*	1.42–34.42	5.27	0.54–51.41
	≥50	9.27***	5.90–14.56	20.83***	4.51–96.30	22.67**	2.65–194.03
Gender (Men)	Women	1.04	0.75–142	2.48**	1.24–4.94	2.13	0.90–5.04
Educational level (University)	None	2.08*	1.09–3.96	0.74	0.16–3.51	0.34	0.06–1.79
	Primary	1.83	0.99–3.38	0.95	0.21–4.38	0.23	0.04–1.22
	Intermediate	2.01*	1.06–3.84	0.71	0.15–3.41	0.33	0.06–1.74
	Secondary	1.60	0.74–3.42	0.29	0.04–2.08	0.30	0.04–2.18
Doctor visit in previous year (0)	≥ 1			2.16*	1.09–4.32	2.57*	1.05–6.28

*p<0.05

**p<0.01

***p<0.001.

This table shows the results of three binary logistic regressions. The first one predicted HTN by age, gender, and educational level. The second predicted HTN awareness by age, gender, educational level, and doctor visit in the previous year. Finally, the last one predicted HTN treatment by the same variables.

### General and central obesity

In terms of BMI, the prevalence of underweight, overweight and general obesity were 12.6% (95% CI: 10.5–14.7), 19.2% (95% CI: 16.7–21.7) and 9.7% (95% CI: 7.9–11.5) respectively. Using WC, the prevalence of central obesity was 26% (95% CI: 23.3–28.7), whereas using WHtR, this prevalence was 39.8% (95% CI: 36.7–42.9). Among men and women, the prevalence of general and central obesity rose with age, and in each age group, women were more often obese (in terms of BMI, WC, and WHtR) than men (Table 3).

**Table 3:** Age- and gender-specific prevalence (%) of underweight, overweight, general obesity and central obesity.

Age	Gender	N	Obesity based on BMI*				Obesity based on WC‡		Obesity based on WHtR^•^	
			Underweight	Overweight	Obese	P	Obese	P	Obese	P
20–29	Female	206	18	16.5	5.8	<0.01	23.8	<0.001	30.6	<0.001
	Male	207	19.3	9.2	1.4		1.4		4.8	
30–39	Female	135	5.9	31.9	16.3	<0.001	49.6	<0.001	69.6	<0.001
	Male	131	16.8	13	3.1		1.5		16.8	
40–49	Female	78	1.3	24.4	29.5	<0.001	67.9	<0.001	84.6	<0.001
	Male	78	9	16.7	3.8		6.4		34.6	
≥ 50	Female	71	2.8	33.8	33.8	<0.001	93	<0.001	95.8	<0.001
	Male	78	9	25.6	5.1		14.1		53.8	

*General obesity was defined as body mass index (BMI) ≥ 30 kg/m² and overweight as 25 ≤ BMI < 30.

‡Central obesity based on waist circumference (WC) was defined as WC ≥ 102 cm for men and ≥ 88 cm for women.

^•^Central obesity based on waist-to-height ratio (WHtR) was defined as WHtR > 0.5.

The binary logistic regression models confirm that age and gender were the primary risk factors for general and central obesity in Dakar (Table 4). Educational level was a significant predictor for central obesity (both WC and WHtR), but not for general obesity.

**Table 4:** Adjusted Odds Ratio (OR) for general and central obesity.

Variables	Categories	General obesity (N = 984)		Central obesity by WC (N = 984)		Central obesity by WHtR (N = 984)	
		OR	CI (95%)	OR	CI (95%)	OR	CI (95%)
Age (20–29)	30–39	2.91**	1.47–5.74	2.89***	1.82–4.60	4.6***	3.05–6.93
	40–49	5.89***	2.92–11.88	7.47***	4.24–13.18	12.03***	7.24–20.00
	≥50	7.20***	3.55–14.57	29.51***	14.79–58.90	28.67***	16.46–49.92
Gender (Men)	Women	7.72***	4.23–14.09	49.33***	26.74–91.01	10.98***	7.54–15.99
Educational level (University)	None	1.22	0.42–3.49	1.43	0.65–3.16	1.59	0.86–2.94
	Primary	1.51	0.55–4.15	2.58*	1.24–5.40	1.66	0.94–2.92
	Intermediate	1.82	0.63–5.23	2.58*	1.17–5.68	2.01*	1.10–3.68
	Secondary	1.9	0.58–6.24	2.91*	1.17–7.21	1.27	0.61–2.64

General obesity was defined as body mass index (BMI) ≥ 30 kg/m²; central obesity based on waist circumference (WC) was defined as WC ≥ 102 cm for men and ≥ 88 cm for women; central obesity based on WHtR was defined as WHtR > 0.5.

*p<0.05

**p<0.01

***p<0.001.

This table shows the results of three binary logistic regressions. The first one predicted general obesity by age, gender, and educational level. The second predicted central obesity (WC) by the same variables. The last one predicted central obesity (WHtR) by the same variables.

### Relationship between hypertension and obesity

**Fig 3:**
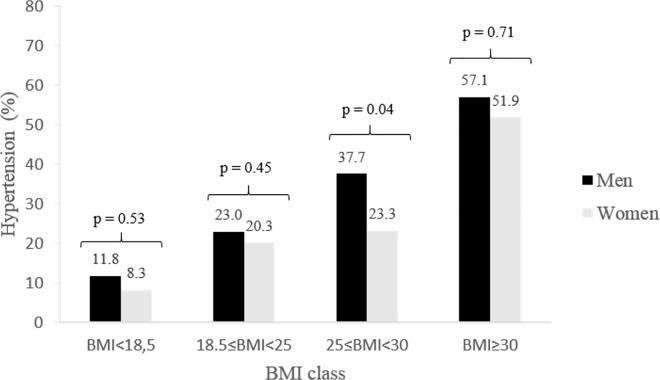
Prevalence of hypertension by BMI.

The prevalence of HTN gradually rose with BMI among men and women (Fig 3). Using WC, 57.1% of obese men were hypertensive whereas only 22.8% of their non-obese counterparts had high blood pressure (p<0.001). Similarly, 36.6% of women with central obesity by WC were hypertensive versus 14.5% for non-obese (p<0.001). Using WHtR, 52.5% and 35.4% of obese men and women were hypertensive whereas only 17.0% and 10.1% of their non-obese counterparts had high blood pressure (p<0.001).

Binary logistic regressions were performed to test the association of HTN with general obesity on the one hand, and central obesity–by WC and WHtR–on the other. Binary logistic regression models showed that after adjustment for age and educational level, general obesity and central obesity by WHtR were significant predictors of HTN among men and women, but central obesity by WC was not (Table 5). More precisely, WHtR was the best predictor of HTN among men, and BMI the best predictor among women. In the total sample–when adjusted for age, gender and educational level–, the odds ratio for general obesity was 3.02 (p<0.001). The same analysis with central obesity by WC and WHtR showed odds ratios of 1.75 (p<0.05) and 2.79 (p<0.001) respectively (Table 5). Neither general obesity nor central obesity was significantly associated with HTN awareness and treatment.

**Table 5:** Associations of hypertension with general and central obesity, adjusted for age and educational level (and gender for the total sample analysis).

Variables	Categories	Total sample (N = 984)		Men (N = 494)		Women (N = 490)	
		OR	CI (95%)	OR	CI (95%)	OR	CI (95%)
BMI (non obese)	Obese	3,02***	1,85–4,93	3,7*	1,16–11,76	2,73***	1,55–4,80
WC (non obese)	Obese	1,75*	1,13–2,72	2,47	0,93–6,55	1,32	0,77–2,26
WHtR (non obese)	Obese	2,79***	1,88–4,15	3,76***	2,21–6,39	2,02*	1,10–3,73

*p<0.05

**p<0.01

***p<0.001.

This table shows the results of nine binary logistic regressions: three for the general sample (men and women), three for the male sample, and three for the female sample. For the general sample, the first logistic regression predicted HTN by age, gender, educational level, and BMI. The second predicted HTN by the age, gender, educational level, and WC. The last one predicted HTN by age, gender, educational level, and WHtR. The same analyses were performed for the male and female samples (without the gender variable into the model). For a clearer reading of the results of interest, only the OR for BMI, WC, and WHtR were noted in the table.

## Discussion

The epidemic of non-communicable diseases, including CVD, is the largest we have ever known in the world [49], and our study confirms that SSA is not immune to this phenomenon. The prevalence of HTN in Dakar (24.7%) corresponded with that observed among people in other sub-Saharan African populations [14, 16–20], and was relatively close to those observed in the United States [24]. Together with the relationship between HTN and educational level, the high prevalence of HTN in our sample seems to indicate that the Dakar population is currently in an advanced stage of epidemiological transition.

However, the problem of HTN in Dakar–and in SSA in general–appears in a very different light compared to developed countries. Indeed, while the more developed countries such as the United States for example [24] have largely surpassed the Rule of Halves, it seems still well out of reach for the Dakar population. Among hypertensive individuals in Dakar, just over 28% were aware of their condition. Among them, over 55% reported taking treatment for high blood pressure, but fewer than 30% of the people reporting treatment had controlled blood pressure, or less 5% of the hypertensive population. In comparison, this rate was over 50% in the United States in 2011–2012 [24]. While improving awareness of HTN in Dakar is a first step toward reducing CVD morbidity and mortality, inability to afford medicine consistently may hinder HTN control efforts in a country where the permanent economic crisis often hampers access to prescribed medication [47].

As observed in (all) other populations [16–20], the prevalence of HTN increased with increasing age in Dakar–an alarming result with respect to the rapid aging of African populations [50]. In Senegal, recent studies tend to indicate that HTN now affects rural populations in similar proportions than what has been observed in Dakar [51]. All these findings and projections underscore the urgent need to develop national strategies for prevention and treatment of HTN in Senegal. Improving medical supervision is the priority direction to follow, as our results indicate that individuals who saw a doctor within the previous year were 2.5 times more likely to be treated for high blood pressure than those who had not seen a doctor.

Although the prevalence of general and central obesity seems fairly low in our sample, it masks major gender and generation disparities. Among women, prevalence rates for general and central obesity by WC were 6 and 11 times higher respectively than those observed among men. Differences in physical activity between men and women, associated with the value placed on plumpness in Senegalese women–as a symbol of peace and wealth in the household–partly explains these gender differences [52,53]. In both sexes, general obesity rose steadily with age, in particular rising from 8.7% among young women to over 67% among older women. Among women over 50, the central obesity rate reached 93% by WC, and 96% by WHtR. These extremely high obesity rates among older women can and should be seen in relation to their revered social status within the family, usually an extended family, which releases them from daily physical activities and leaves older adults first choice from the common pot [54, 55]. However, the thresholds for WC and WHtR are perhaps not appropriate for this population. This question warrants further study in Senegal.

In our sample, only central obesity was associated with educational level: individuals at the three intermediary levels were significantly more frequently obese than the better educated, but not the less educated. This result differs from those obtained in Europe and in the USA where educational inequalities are related with increased BMI, people with lower educational levels being more likely to be obese [56,57]. This result indicates that the epidemiological transition–characterized by the transfer of chronic disease risk factors from more educated people in the early stages of the process to the least educated toward the end of the transition [58]–is currently at an advanced stage in Dakar, but not entirely completed.

Lastly, general obesity and central obesity were significantly associated with HTN in our entire sample, even when these correlations were controlled for socio-demographic factors. It is well known that a large part of the incidence of HTN is directly related to obesity. In Dakar, the relationship between HTN and general obesity (OR = 3.02; p<0.001) was stronger than the relationship between HTN and central obesity by WC (OR = 1.75; p<0.05). This has already been observed in other populations [34,36]. But in Dakar, considering the sexes separately, we noted that central obesity by WC was not significantly associated with HTN. WHtR was the best predictor of HTN among men (OR = 3.76; p<0.001), and BMI the best predictor among women (OR = 2.73; p<0.001). Indeed, this study confirms the relevance of analyzing WHtR as a predictor of cardiometabolic risk in SSA [44].

Our investigation has several limitations. First, our study design was cross-sectional, which does not allow us to explore causation. To overcome this limitation, it would be necessary to conduct a longitudinal study in Dakar in the future. Second, as in many studies, arterial blood pressure was measured twice during a single visit, which may lead to misestimating the prevalence of HTN due to conditions such as white-coat HTN and masked HTN [59]. Third, the HTN treatment rate was assessed solely by individual self-reporting. The social desirability bias regarding treatment reporting among those aware of HTN is of course a limitation that verification of the actual presence of medication in the home might have limited.

In conclusion, this study has demonstrated a high prevalence of HTN in Dakar. The awareness, treatment, and effective control of HTN are unacceptably low. Improving awareness of HTN in Dakar is critical to reducing and preventing morbidity and mortality from CVD. Obesity is also highly prevalent among women, and particularly among older women. This phenomenon should be seen in the light of the lack of physical activity among women, cultural reverence of plumpness and the social status of older adults within the family. Finally, HTN was strongly correlated with general obesity among women and with central obesity by WHtR among men. Indeed, the blood pressure of women with general obesity, and of men with central obesity, in the community should be monitored regularly to identify patients with high blood pressure early and limit the CVD burden in Senegal.

## Supporting Information

S1 File Database. (XLSX)
